# Investigation of hindbrain activity during active locomotion reveals inhibitory neurons involved in sensorimotor processing

**DOI:** 10.1038/s41598-018-31968-4

**Published:** 2018-09-11

**Authors:** Kristen E. Severi, Urs L. Böhm, Claire Wyart

**Affiliations:** 1 Institut du Cerveau et de la Moelle épinière, ICM, Sorbonne Université, Inserm, CNRS, AP-HP, F-75013 Paris, France; 2 Present Address: Federated Department of Biological Sciences, New Jersey Institute of Technology, University Heights, Newark, NJ 07102 USA; 3 Present Address: Dept. of Chemistry and Chemical Biology, Harvard University, Cambridge, MA 02138 USA

## Abstract

Locomotion in vertebrates relies on motor circuits in the spinal cord receiving inputs from the hindbrain to execute motor commands while dynamically integrating proprioceptive sensory feedback. The spatial organization of the neuronal networks driving locomotion in the hindbrain and role of inhibition has not been extensively investigated. Here, we mapped neuronal activity with single-cell resolution in the hindbrain of restrained transgenic *Tg(HuC:GCaMP5G)* zebrafish larvae swimming in response to whole-field visual motion. We combined large-scale population calcium imaging in the hindbrain with simultaneous high-speed recording of the moving tail in animals where specific markers label glycinergic inhibitory neurons. We identified cells whose activity preferentially correlates with the visual stimulus or motor activity and used brain registration to compare data across individual larvae. We then morphed calcium imaging data onto the zebrafish brain atlas to compare with known transgenic markers. We report cells localized in the cerebellum whose activity is shut off by the onset of the visual stimulus, suggesting these cells may be constitutively active and silenced during sensorimotor processing. Finally, we discover that the activity of a medial stripe of glycinergic neurons in the domain of expression of the transcription factor *engrailed1b* is highly correlated with the onset of locomotion. Our efforts provide a high-resolution, open-access dataset for the community by comparing our functional map of the hindbrain to existing open-access atlases and enabling further investigation of this population’s role in locomotion.

## Introduction

When exploring their environments, animals constantly perform updates based on their motor actions and available sensory information. In the larval zebrafish, which swim via discrete bouts of tail oscillation^1^, there is still much to learn about the neural circuits which integrate sensory information from the outside world with proprioception, and signal to initiate locomotion. A great challenge in the study of motor control has been the technical difficulty of recording activity from neurons responsible for motor actions, in the midst of active motor actions. The experimental solution to this has mostly been to work in reduced or paralyzed preparations, where proprioceptive signals are either missing or reporting a failure to move. In order to best capture the neural activity representing these processes, functional recordings can be performed during active motion.

While large scale population imaging data can be extremely informative on their own, new tools are emerging to genetically target specific populations of neurons and to register them in brain atlases, with close to cellular resolution^2–4^. By taking advantage of these existing transgenic lines, and with open-access larval zebrafish brain atlases, we have an opportunity to look at the activity of large populations of neurons and place that functional activity in the context of known markers, without the painstaking process of fixing and staining.

Previous work can inform our expectations regarding excitatory (glutamate) and inhibitory (glycine and GABA) neurotransmitter phenotypes in the hindbrain. Kinkhabwala *et al*., revealed an intricate organization of the hindbrain in alternating stripes of neurons based on glutamatergic and glycinergic neurotransmitter expression^5–7^. These stripes reflect an underlying organization of the hindbrain, and are stacked according to developmental age, input resistance, and anatomical properties^5,6^. Yet the functional recruitment of interneurons from these stripes during active swimming is not yet known. GABAergic neurons were not investigated in that work, although we know they are found in large numbers in the hindbrain and cerebellum, previously identified by transgenic lines and *in situ* staining^7–9^.

The medial-most glycinergic hindbrain stripe identified in Kinkhabwala *et al*., has been shown to be positive for the expression of the transcription factor *engrailed1b* (EN1 in mammals)^5,6,10–14^. The only functionally identified neuronal class in the *engrailed1b*^+^ glycinergic stripe are the feedback-inhibitory neurons (FINs), known to provide glycinergic inhibition to the Mauthner cells during a very specific fast type of movement, the escape response^6^. Thus, there is still much left unexplored.

Here we ask which neurons responsible for motor commands and sensorimotor processing are located in or near the zebrafish hindbrain. We use population calcium imaging to record from the brains of actively swimming but restrained larval zebrafish in an attempt to incorporate multisensory signals into our dataset. Since such information processing may incorporate both excitatory and inhibitory cell types, we then ask if these active populations are inhibitory by mapping them onto known transgenic lines in the ZBB atlas^3,4^.

## Results

**Figure 1:**
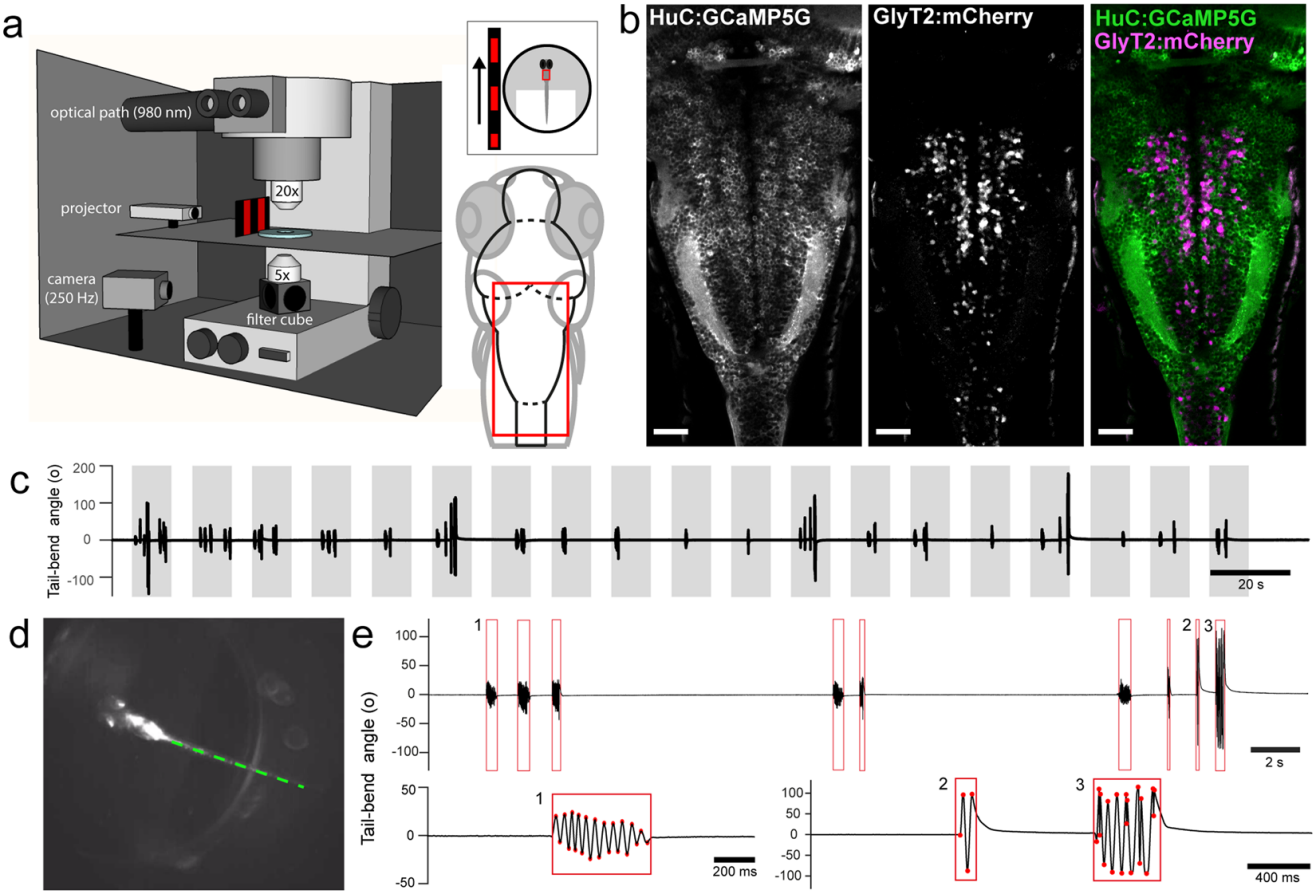
Single-cell resolution calcium imaging of the hindbrain during active visuomotor behavior. (**a**) The experimental set up consists of an *in vivo* 2-photon laser scanning microscope imaging at 980 nm and acquiring at 5.81 Hz while optomotor gratings are projected on a screen on the left or right side of the larva, which is embedded dorsal side up in agarose with the tail freed. A high-speed camera captures the tail movements from below at 250 Hz. The red box indicates the imaging region comprising the majority of the hindbrain. (**b**) Z-projection stack of several optical sections imaged from the dorsal view in *Tg(HuC:GCaMP5G;glyt2:loxP-mCherry-loxP-Gal4; mifta*^−*/*−^*)* transgenic larvae reveals the localization of GCaMP5G (left) in most neurons and mCherry (center) in glycinergic neurons (right, merge with GCaMP5G in green and mCherry in magenta). (**c**) Trials during which a single plane is imaged for 315 s with sequential presentations of moving optomotor stimuli for 10 s in open-loop (grey bars). Larvae initiate locomotion more frequently during the periods of stimulus motion than when the stimulus is stationary. (**d**) Single image extracted from a high-speed behavioral movie showing the larva under IR illumination with its head embedded in agarose and the tail cut free. Offline tracking of the tail in eight sections between the caudal end of the swim bladder and end of the tail. (**e**) Tail-bend angle (degrees) defined between the swim bladder and the end of the tail can be plotted and bouts of swimming are automatically extracted (red boxes). Tracking failures and obvious struggles were excluded from the dataset. Example swim bout expanded below (1) and example struggles expanded below (2,3). Red points mark automatically detected tail deflections. Each detected bout is bounded by a red box. Scale bars are 40 µm in (**b**).

Here we took advantage of the capability to restrain an actively swimming larval zebrafish to record the activity of a large population of hindbrain neurons using calcium imaging during locomotion (Fig. 1a). In order to investigate the role of glycinergic neurons in the hindbrain, we utilized *Tg(HuC:GCaMP5G; glyt2a:mCherry)* double transgenic animals, expressing pan-neuronally the GCaMP5G calcium indicator^15^ with mCherry expressed in glycinergic neurons^9^, homozygous for the *mifta* mutation (*mitfa*^−/−^^16,17^) (Fig. 1b, Methods). By inducing locomotion periodically with optomotor stimuli^18–20^, restrained larvae reliably swam in response to the visual cue (Fig. 1c), and tail movements were recorded simultaneously (Fig. 1d) without spectrally interfering with calcium imaging. Tail movements were analyzed offline (Fig. 1e), the kinematics of which were similar to previous reports^19,21^.

**Figure 2:**
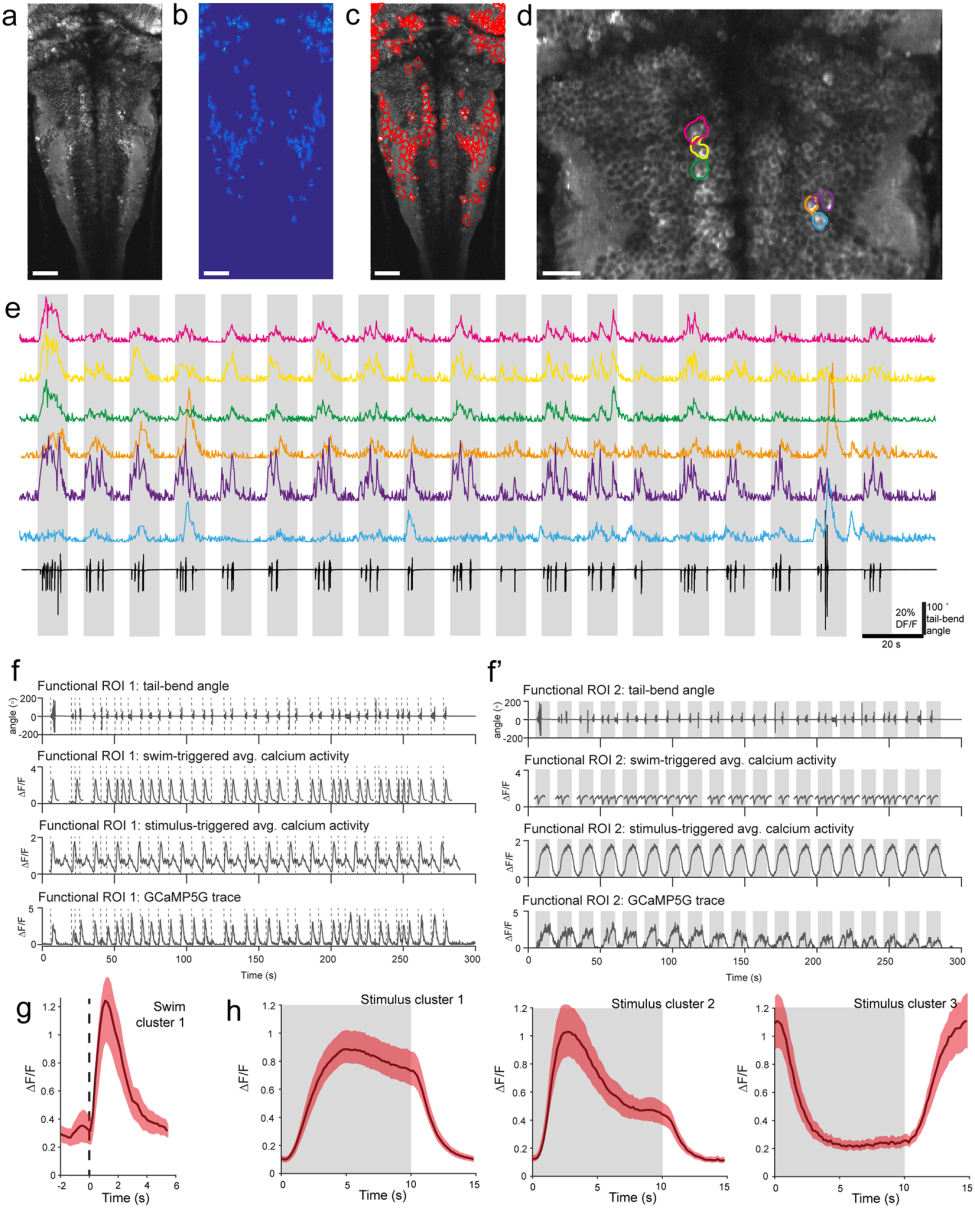
Automatic extraction of calcium signals and clustering analysis reveal different responses to the stimulus presentation as well as recruitment during locomotion. (**a**) Standard deviation of time series obtained from an example plane of calcium imaging data. (**b**) Spatial map of a subset of active regions extracted from that plane. (**c**) Red outlines are borders of automatically detected functional ROIs. (**d**) Zoom on some example regions of interest (ROIs) from the same plane as (**a–c**). (**e**) Six different example ROIs (colors match locations in panel **(d)**) plotted for the full length of the trial. Periods of optomotor stimulus motion are indicated by grey bars. Tail trace is plotted below in black. (**f**) Sample functional ROIs that show activity during visuomotor behavior. From top to bottom: tail-bend angle, swim-triggered average calcium activity, stimulus-triggered average calcium activity, GCaMP5G ΔF/F trace. Individual bout starts are marked with a vertical dotted line. This functional ROI (1) correlates better with swim-events than with the visual stimulus. (**f** ’) Same as (**f** ) but for a different functional ROI (2) which correlates better with the stimulus than with swim events. Periods of optomotor stimulus motion are indicated by grey bars. (**g**) Cluster mean from clustering swimming-related activity. Dotted line indicates bout start. Red shaded area around solid black line (mean) indicates +/−  S.E.M (n = 144 ROIs from 7 larvae). (**h**) Cluster means from clustering stimulus related activity. Grey shaded area indicates when stimulus is moving. Red shaded area around black line (mean) indicates +/− S.E.M (swim cluster 1: n = 466 ROIs from 10 larvae, swim cluster 2: n = 161 ROIs from 10 larvae, swim cluster 3: n = 76 ROIs from 8 larvae). Scale bars are 40 µm in (**a–c**), and 20 µm in (**d**).

To identify regions of activity that correlated with either motor activity or presentation of the visual stimulus agnostic to what this activity would look like, we adopted a two-step approach, similar to Haesemeyer *et al*.^22^. First, we applied an automated calcium source extraction method to identify potentially interesting activity in the calcium imaging data^23^ (Fig. 2a–e). This method results in a fixed number of potentially active regions of interest (ROIs) per analyzed imaging plane. To identify those ΔF/F traces that most strongly correlated with either motor activity or visual stimulus presentation, we correlated each ΔF/F trace to its swim or stimulus-triggered average (Fig. 2f-f’). Calcium activity that reliably changes with motor activity should correlate well with its swim-triggered average but not with its stimulus-triggered average and the opposite should hold true for calcium activity elicited by the visual stimulus (Supplementary Fig. S1). In order to identify cells active during stimulus presentation or swimming, we selected ΔF/F traces with a high correlation with either their stimulus-triggered average or swim-triggered average, but not both (Supplementary Fig. S1). To classify patterns of activity amongst these ΔF/F traces, we used an unbiased method and clustered the stimulus and swimming evoked groups separately^24^. We identified four different clusters of activity, one from the group of ROIs that are active during swimming (Fig. 2g, Supplementary Video S1) and three more from the group of ROIs that correlated to the moving visual stimulus (Fig. 2h, Supplementary Videos S2–4). Stimulus cluster 1 shows increased activity during stimulus presentation while stimulus cluster 3 shows decreased activity during stimulus presentation (Fig. 2h). The stimulus cluster 2 appears as a mixture between stimulus-evoked and motor-related activity as can be judged from the activity profile that is intermediate between swimming cluster 1 (Fig. 2g) and stimulus cluster 1 (Fig. 2h). Such a mixture may arise from the inherent high correlation between stimulus presentation and swimming activity in our assay. Consequently, the correlation of a trace with its stimulus-triggered average is not independent from the correlation with its swim-triggered average.

We then used the identified cluster means as regressors to identify the brain regions in each recording showing activity correlated to any of the clusters. We therefore calculated the pixel-wise correlation of each cluster mean during each swim event (for swim cluster 1) and each stimulus presentation (for stimulus clusters 1–3). To compare activity related to each cluster between larvae and brain regions, we registered the resulting average correlation maps for all 10 larvae to a common reference brain. Using *Tg(vglut2a:DsRed)* expression as a bridge, we registered our dataset with the ZBB brain atlas^3,4^. Once this bridge was established, we were able to easily compare the pixels associated with the clusters identified above with the transgenic lines available in the ZBB atlas.

**Figure 3:**
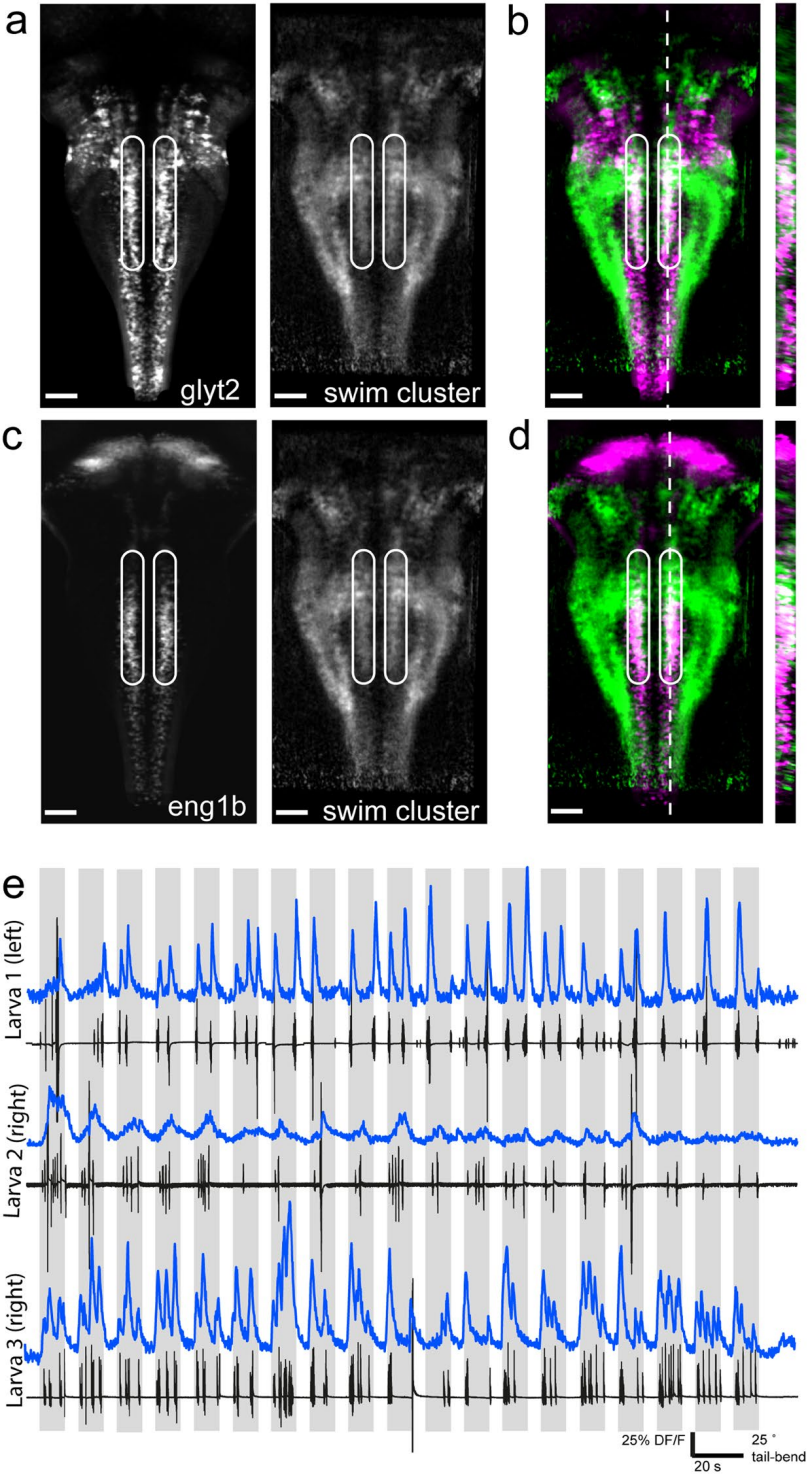
Recruitment of a glycinergic and *engrailed1b*-positive stripe during locomotion. (**a**) Z-projection stack of 30 frames from our processed dataset registered with the ZBB atlas. Left: ZBB atlas stack of *Tg(glyt2:GFP)* expression; right: pixels correlated with swim cluster 1 (weighted average across larvae). Area of interest bounded by white circles. (**b**) Overlap of the two images in (**a**) with *Tg(glyt2:GFP)* shown in magenta and swim cluster 1 shown in green. Area of interest bounded by white circles. Resliced image at right from position of white dotted line. (**c**) Z-projection stack of 30 frames from our processed dataset registered with the ZBB atlas. Left: ZBB atlas stack of *Tg(engrailed1b:Gal4;UAS:Kaede)* expression; right: pixels correlated with swim cluster 1 averaged across all larvae. Area of interest bounded by white circles. (**d**) Overlap of the two images in (**c**) with *Tg(engrailed1b:Gal4;UAS:Kaede)* shown in magenta and swim cluster 1 shown in green. Area of interest bounded by white circles. Resliced image at right from position of white dotted line. (**e**) Example traces from three different larvae showing calcium activity from a single, manually draw ROI located within the area of interest above on one side of the midline (left or right, indicated). ΔF/F trace shown above in blue and the accompanying tail trace in black for the example trial. Grey-shaded area indicates when stimulus is moving. Scale bars are 40 µm for (**a–d**).

We found considerable spatial overlap between regions that correlate with the swim cluster and the images of the *Tg(glyt2:mCherry)* line from our own dataset, and also with the images of the *Tg(glyt2:GFP)* line in the ZBB atlas, indicating activation of glycinergic cells during swimming (Fig. 3a,b). This was unexpected because few hindbrain glycinergic neurons have been implicated in locomotion thus far, and the specific cells which were previously identified were circuit elements in the complex and specific escape response rather than standard swimming^6^. The spatial pattern of overlap we observed was localized to one particular region: the medial-most glycinergic stripe described by Kinkhabwala *et al*.^5,6^, By comparing the overlap between our swim cluster and the ZBB transgenic line *Tg(engrailed1b:Gal4; UAS:Kaede)* we concluded the swim cluster resides in the *engrailed1b*^+^ glycinergic stripe in the hindbrain (Fig. 3c,d, Fig. S2, Supplementary Video S5). This cluster is active during locomotor bouts and is extremely well locked to swimming (Fig. 3e). Note that the temporal resolution of our calcium imaging acquired at 5.81 Hz does not allow discrimination of recruitment at the onset or offset of locomotor bouts specifically. Due to the large number of cells in this cluster spanning several rhombomeres, we conclude that there are likely double-positive *glyt2*^+^ and *engrailed1b*^+^ neurons beyond the previously identified FINs^6^. FINs are characterized to have a short axon and are located in close proximity to the Mauthner cell^6^. The identity and contribution to movement coordination by this large population of ipsilaterally-ascending hindbrain neurons remains to be explored in detail.

**Figure 4:**
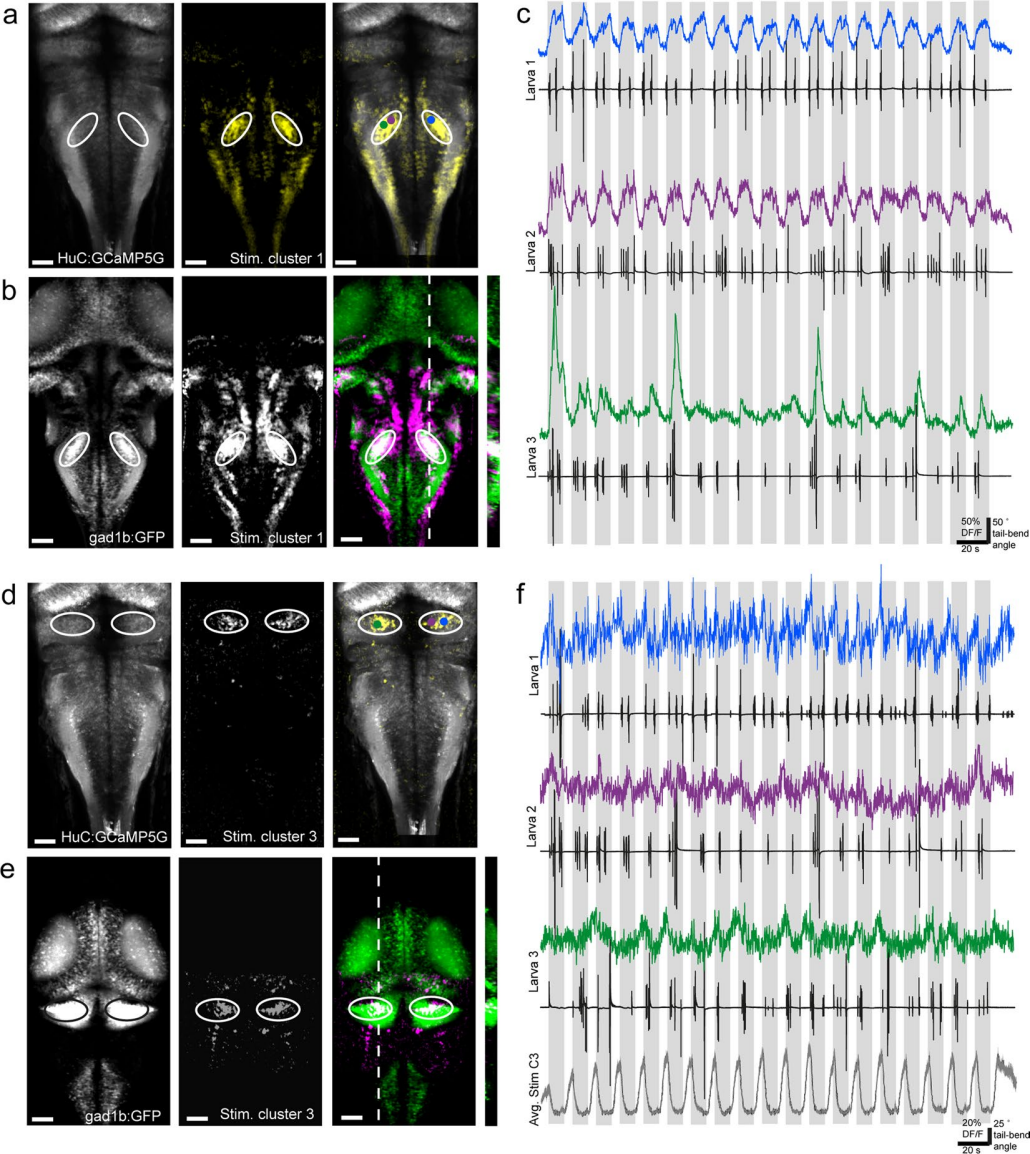
Different GABAergic clusters in hindbrain and cerebellum show activity either positively or negatively correlated with the visual stimulus. (**a**) Z-projection stack of 20 optical sections imaged from the dorsal aspect of *Tg(HuC:GCaMP5G)* larvae compared with stimulus cluster 3. Panels left to right: *Tg(HuC:GCaMP5G)* larvae showing pan-neuronal localization of GCaMP5G; pixels correlated with stimulus cluster 3 averaged across all larvae; merge with HuC:GCaMP5G in grey and stimulus cluster 3 pixels in yellow. Area of interest is bounded by white circles. Colored dots represent ROIs plotted in (**c**). (**b**) Z-projection stack of 26 frames from stimulus cluster 3 correlated pixels registered with the ZBB atlas for the same area of interest as (**a**). Panels left to right: ZBB atlas stack of *Tg (gad1b:GFP)* expression; pixels correlated with stimulus cluster 3 averaged across all larvae; overlap of the two images with *Tg(gad1b:GFP)* shown in green and stimulus cluster 3 shown in magenta. Area of interest is bounded by white circles. Resliced image at right from position of white dotted line. (**c**) Example traces from three different larvae showing calcium activity from an ROI located within the area of interest outlined in white in panels (**a**,**b**). ΔF/F traces are shown in color matching the ROI centroid’s approximate location on panel (**a,** right). The accompanying tail traces for that individual trial are shown in black. Grey-shaded area indicates when stimulus is moving. (**d**) Z-projection stack of 25 optical sections imaged from the dorsal view in *Tg(HuC:GCaMP5G)* larvae compared with stimulus cluster 1. Panels left to right: *Tg(HuC:GCaMP5G)* larvae showing pan-neuronal localization of GCaMP5G; pixels correlated with stimulus cluster 3 averaged across all larvae; merge with HuC:GCaMP5G in grey and stimulus cluster 3 pixels in yellow. Area of interest is bounded by white circles. Colored dots represent ROIs plotted in (**f**). (**e**) Z-projection stack of 28 frames from stimulus cluster 3 correlated pixels registered with the ZBB atlas for the same area of interest as (**d**). Panels left to right: ZBB atlas stack of *Tg (gad1b:GFP)* expression; pixels correlated with stimulus cluster 3 averaged across all larvae; overlap of the two images *Tg(gad1b:GFP)* shown in green and stimulus cluster 3 shown in magenta. Area of interest is bounded by black or white circles. Resliced image at right from position of white dotted line. (**f**) Example traces from three different larvae showing calcium activity from an ROI located within the area of interest outlined in panels (**d**,**e**). ΔF/F traces are shown in color matching the ROI centroid’s approximate location on panel (**d**, right). The accompanying tail traces for that individual trial are shown in black. Below the average ΔF/F trace for pixels associate with stimulus cluster 3 with shading indicating +/− S.E.M (n = 76 ROIs from 8 larvae). Grey-shaded vertical bars indicate when visual stimulus is moving. Scale bars are 40 µm for (**a**,**b**) and (**d**,**e**).

Another interesting group of cells identified by clustering was included in the first stimulus-related cluster, where activity was high and prolonged during stimulus motion (Fig. 2h, left). The most prominent regions of stimulus cluster 1-associated pixels were large, bilaterally paired, oval regions in the widest part of the hindbrain (Fig. 4a). When we compared the stimulus cluster 1 data in this region to the ZBB atlas we found the identical oval-shaped regions in the *Tg(gad1b:GFP)* transgenic line which co-localize (Fig. 4b). The activity of functional ROIs in these regions are highly stimulus-locked (Fig. 4c), although in some cases it reflects the tail-bend amplitude (Fig. 4c, bottom trace). It would be very interesting to know the anatomy of cells in the GABAergic nuclei identified here, and whether these cells project locally or over a long distance. Future electrophysiological characterization of these cells will be critical to determining their temporal recruitment both during visually-induced locomotor events and during visual stimulation which does not elicit movement.

A cluster with a nearly inverse profile to stimulus cluster 1 is stimulus cluster 3, where activity appeared to be suppressed during the presentation of the stimulus when the stimulus is moving (Fig. 2h, right). The cluster 3 was much smaller than the other clusters in terms of assigned pixels (compare Videos S1–4). Interestingly, the vast majority of pixels where activity is reduced during stimulus motion were located in the cerebellum (Fig. 4d, Supplementary Fig. S3). At the developmental stage when these experiments were performed (5–6 dpf), the three-layer structure of the cerebellum is already established^8,25^, and both glutamatergic and GABAergic cells are active^21,26–31^. Using the ZBB atlas, we found substantial overlap with *Tg(gad1b:GFP)* (Fig. 4e), which labels Purkinje, Golgi, and stellate cells in the zebrafish cerebellum^8,25^. The observed suppression of calcium activity may reflect a decrease in baseline activity in GABAergic cerebellar neurons during the stimulus presentation (Fig. 4f). GABAergic Purkinje cells have been studied at larval stages (specifically 4–8 dpf) and exhibit heterogeneous and highly variable activity including simple and complex spiking, and various tonic and bursting states^27,28,31^. A suppression of Purkinje cell activity in response to any visual stimulation has rarely been reported^31^. A combination of electrophysiology with whole cerebellum calcium imaging will be necessary to characterize cells from this cluster.

## Discussion

Our study provides the community with an open-access, single cell-resolution population imaging dataset of hindbrain calcium activity during active locomotion induced by optomotor stimuli. Here we investigated which inhibitory neurons are recruited during sensorimotor integration and found interesting responses in diverse populations of glycinergic and GABAergic neurons whose activity correlates with motor generation and/or visual integration.

In order to identify the neurotransmitter phenotypes of functional clusters, we relied on the combination of transgenics as well as the use of the recent creation of open-access zebrafish larval brain atlases by several groups^2–4,32^. These larval brain atlases have enabled the integration of functional datasets with anatomical markers, in both fixed and live brains. By using a combination of live transgenic markers and registering with the ZBB atlas, we found an anatomical stripe of glycinergic neurons recruited during locomotion. Previous work on glycinergic contributions to zebrafish locomotion has shown that mutants and pharmacological blocks of glycine receptors and transporters cause locomotor defects, although these global methods are not able to target specific regions of the brain and spinal cord^33–37^. Anatomical characterizations of glycinergic neurons in the brain across development have revealed both a striped pattern of organization and a heavy innervation of the Mauthner cells by glycinergic neurons, implicating them in the regulation of the escape response^5–7,38,39^.

A recent paper in mammals has brought the role of glycinergic neurons in the brainstem into renewed focus^40^. Capelli *et al*., revealed the distinct roles of glutamatergic and glycinergic interneurons within brainstem paragigantocellular nuclei, which could not be resolved by activating the entire anatomical nucleus. Only by separating these intermixed populations did neurotransmitter-specific activation produce locomotor responses: either high-speed initiation of locomotion or full stop. These observations highlight the need for studies deciphering the opposing functions of neighboring cells of differing neurotransmitter phenotype and might explain contradicting observations from deep brain electrical stimulation in humans and macaques^41,42^. Capelli *et al*., specifically found that optogenetic activation of glycinergic cells of the lateral paragigantocellular nucleus induced locomotor halt or speed reduction proportional to stimulation laser intensity. It is an enticing possibility that a portion of the zebrafish hindbrain glycinergic stripe may represent a basal homologous structure common to vertebrates, although further functional, molecular, and anatomical characterization will be necessary.

The swim cluster 1 region we identify appears to co-localize with the medial-most glycinergic stripe, which is *engrailed1b*^+^. The engrailed transcription factor has long been studied across vertebrates for its role in development of particularly the midbrain-hindbrain boundary and the spinal cord^11,43–45^. In the spinal cord, where *engrailed*^+^ neurons have been best characterized, they perform a critical role providing ipsilateral ascending inhibition across vertebrate species^12–14,46^. In the mammalian midbrain, *engrailed1* has been implicated in a role in Parkinson’s disease^47,48^. Apart from the previously identified FIN neurons^6^, also ipsilateral and ascending, the role of the vast majority of *engrailed1b*^+^ hindbrain neurons has not been explored in zebrafish. In this study, we find activation of these neurons concurrent with locomotion. Future work should attempt to disentangle the various types of locomotor actions, whether it be comparing swimming to turning, or various swimming speeds, in order to further clarify whether *engrailed1b*^+^ neurons participate in all forms or specific forms of motor output. Intersectional genetic approaches in the future will allow disambiguation of midbrain-hindbrain *engrailed1b*^+^ neuronal function from their spinal counterparts.

Further investigation of the neuronal populations we identified here with electrophysiology or systematic single-cell ablation methods, combined with whole brain imaging, will elucidate their precise contribution to movement coordination. The temporal kinetics of voltage indicators^49–51^, faster imaging techniques such as light-sheet imaging^52–56^, combined with cell-targeted electrophysiology will most likely be instrumental to resolve the recruitment of *engrailed1b*^+^ cells relative to the start and end of movement. The combination of light-sheet imaging and electrophysiological techniques will give the community a great opportunity to further explore the role of feedback in active circuits.

## Materials and Methods

### Animal care and transgenic lines utilized

All procedures were approved by the Institut du Cerveau et de la Moelle épinière (ICM) and the National Ethics Committee on E.U. legislation (Comité National de Réflexion Ethique sur l’Expérimentation Animale) based on EU legislation or were in accordance with institutional and national guidelines. Larvae were raised in an incubator set to 28.5 °C under a 14/10 light/dark cycle. Experiments were performed at room temperature (22–26 °C) on 5–6 days post-fertilization (dpf) larvae. All experiments were performed on *Danio rerio* larvae of AB, TL, or mixed background of *mitfa* -/- genotype (nacre^16,17^). Triple transgenic animals were *Tg(elavl3:GCaMP5G; glyt2a:loxP-mCherry-loxP-Gal4;UAS:GFP*,*cmlc2:GFP*) referred to as *Tg(HuC:GCaMP5G; glyt2:mCherry)* for simplicity throughout, by crossing *Tg(elavl3:GCaMP5G)*^15^ with *Tg(glyt2a:loxP-mCherry-loxP-Gal4;UAS:GFP;cmlc2:GFP)*^9^ and screening for double-positive embryos. Additionally, transgenic *Tg(vglut2a:loxP-DsRed-loxP-GFP)*^6^ larvae referred to as *Tg(vglut2a:DsRed)* were used for comparing and bridging datasets.

### Calcium imaging and behavior setup

Double transgenic *Tg(HuC:GCaMP5G; glyt2:mCherry)* larvae were mounted in glass-bottom dishes (MatTek, Ashland, MA, USA) filled with 1.5% low-melting point agarose, and the agarose surrounding the tail was removed. Calcium imaging was performed on a two-photon microscope (2p-vivo, Intelligent Imaging Innovations, Inc., Denver, CO, USA) including a Mai Tai Deepsee laser (Spectra-Physics) tuned to 980 nm and a Zeiss Axio-Examiner Z1 with a Zeiss W Plan-APO 20X water-immersion objective. Fluorescent images were acquired using Slidebook software (3i Intelligent Imaging Innovations, Inc., Denver, CO, USA) at 5.81 Hz with 1.2 µs dwell time during scanning. Each trial collected in a single plane was 315 s in duration and the image size was 512 × 249 pixels. Single-plane acquisitions were spaced by 10 µm in z and collected from dorsal to ventral in the brain. Optomotor stimuli were presented using an MP160 pocket projector (3 M) filtered with a Wratten #29 deep red filter (long pass filter with 50% transmission at 620 nm, Kodak, USA). The stimulus was created using Labview 2012 (National Instruments) and consisted of a square-wave grating alternating between 10 s of motion in a caudal-to-rostral direction with 5 s static presentation, delivered to a screen positioned on one side of the animal. To record tail movement, a similar setup was used in Bӧhm, Prendergast *et al*., 2016^57^ where an 890 nm LED was used to illuminate the larva and behavior was recorded at 250 Hz from below through a 5x microscope objective and imaged on a camera (Basler) with a 50 mm camera objective. Infrared light from the imaging laser was blocked with a filter located in front of the camera. Calcium imaging, behavior, and stimulus presentation were synchronized via a Digidata series 1322 A digitizer, and pClamp 8.2 software (Molecular Devices–Axon Instruments, Sunnyvale, CA, USA). After each experiment, the larva was anesthetized with MS-222 and a high-resolution Z-stack was recorded with 1 µm z-step for both red (mCherry) and green (GCaMP5G) channels simultaneously. Parameters were: 1024 × 499–1024 pixels, 10 pixel averaging, 3–5 frame averaging, 4–8 µs dwell time per pixel.

### Analysis of behavioral videos and bout extraction

Using custom MATLAB software (Mathworks, Natick, MA, USA), 8 segments of the tail were extracted between manually chosen points at the end of the swim bladder and the tip of the tail^19,57,58^. From the tail-bend angle over time, we detected local maxima and minima from which bout and interbout durations were extracted. All bouts were visually inspected by experimenters and bouts were manually excluded from the dataset in cases of tracking failures, or when belonging to obvious escape or struggle behavior. Bouts were not further subdivided or categorized for this analysis. All custom MATLAB scripts are available upon request.

### Image processing and ROI extraction

Image time series were motion corrected in 2D using fast non-rigid motion correction (NoRMCorre)^59^. Initial ΔF/F fluorescence traces were extracted using the automated source extraction methods and code published in Pnevmatikakis *et al*., 2016^23^ (Parameters: Number of components: 150, standard deviation of Gaussian kernel: 5, merge threshold: 0.97). After testing a range of number of components with our data, the algorithm always selected less than 150 meaningful components, thus we selected 150 to ensure none were missed. This method resulted in 150 traces per trial, without verifying that all of them contain significant activity.

To define the traces whose activity was most locked to either stimulus presentation or the occurrence of a swim bout, we used the following two-step approach. First, we extracted the parts of the trace around each swim bout (2 s before and 5 s after the start of a swim bout and >0.5 s after a previous bout) and around each stimulus presentation (5 s before and the full 10 s during which the stimulus is moving) in order to calculate the average swim-triggered and stimulus-triggered traces respectively. Second, we calculated the average correlation coefficient of each individual swim-triggered and stimulus-triggered trace to its swim- and stimulus-triggered average respectively.

We used this measurement to evaluate how consistently the calcium signal changed during either swimming or stimulus presentation and to categorize each trace as swim-driven or stimulus-driven. Traces were defined as stimulus-driven when having an average correlation coefficient >0.6 with their stimulus-triggered average and swim-driven when having and average correlation coefficient >0.6 with their swim-triggered average. Traces with an average correlation coefficient >0.6 with both their swim-triggered and stimulus-triggered average were considered ambiguous and excluded from the dataset (Supplementary Fig. S1).

### Clustering and anatomical mapping

To identify different clusters of activity within each of these categories, the previously identified average swim-triggered traces and stimulus-triggered traces were then clustered for each category independently. This allowed us to define cluster centroids that correlate either with the occurrence of swim bouts or the presentation of the visual stimulus. Clustering was based on methods published in Bianco *et al*., 2015^24,60^ (correlation threshold was 0.75, minimum number of functional ROIs per cluster was 50). To map the occurrence of each identified clusters back to the original anatomical stack, we extracted the swim-triggered and stimulus-triggered frames from the original calcium imaging time series. Each frame was filtered with a 2 pixel mean spatial filter before performing pixel time series correlation to the swim-triggered clusters and stimulus-triggered clusters respectively. The individual correlation maps for each cluster were then averaged over all swim bouts and stimulus presentations to obtain the final average correlation maps. For a schematic representation of this process please refer to Supplementary Fig. S4.

### Brain registration

Brain registration was performed using the Computational Morphometry Toolkit (CMTK, http://nitrc.org/projects/cmtk) and registration parameters were selected based on Marquart *et al*., 2015^3^. High-resolution stacks of the GCaMP5 expression of each brain were registered to a common reference brain using affine registration (parameters:–dofs 12–min-stepsize 1–max-stepsize 32, Supplementary Video S6). A high resolution recording of a *Tg(HuC:GCaMP5G; vglut2a:DsRed)* brain served as the reference. To reduce a low level of bleed-through of the DsRed fluorescence into the GCaMP5G channel, a bleed-through factor was estimated from the scatter plot of pixel values of the DsRed channel vs. the GCaMP5G channel. The pixel values in the GCaMP5G channel were then reduced by the value of the same pixel in the DsRed channel times the bleed-through factor.

To register the functional data to the reference brain, the average intensity of each calcium imaging time series was concatenated to generate a stack. These volumes were then registered to the high-resolution stack of the same brain using warp registration (affine parameters:–dofs 12–min-stepsize 1–max-stepsize 32, warp parameters:–fast–grid-spacing 100–smoothness-constraint-weight 1e-1–grid-refine 2–min-stepsize 0.25–adaptive-fix-thresh 0.25). The resulting transformations were used to register all correlation maps emanating from a given larva to the reference brain to compare anatomical location across larvae. To bridge our functional and anatomical data to publicly available z-brain-browser data (Marquart *et al*., 2016), the recording of the DsRed in the *Tg(vglut2a:DsRed)* from our reference brain was used to perform affine registration to the DsRed in the *Tg(vglut2a:DsRed*) larvae from the ZBB reference brain (parameters:–dofs 12–min-stepsize 1–max-stepsize 32, Supplementary Video S7). This ultimately allowed us to bridge and load any of our data into the ZBB and compare with transgenic lines found in the atlas (Supplementary Videos S8–9). For a schematic representation of this process please refer to Supplementary Fig. S4.

## Electronic supplementary material

Swim cluster 1 pixels (green) and HuC:GCaMP5G (magenta) moving dorsal to ventral.

Stimulus cluster 1 pixels (green) and HuC:GCaMP5G (magenta) moving dorsal to ventral.

Stimulus cluster 2 pixels (green) and HuC:GCaMP5G (magenta) moving dorsal to ventral.

Stimulus cluster 3 pixels (green) and HuC:GCaMP5G (magenta) moving dorsal to ventral.

Swim cluster 1 pixels (green) and engrailed1b (magenta) moving dorsal to ventral.

HuC:GCaMP5G averaged over all larvae (green) registered to our reference brain HuC:GCaMP5G stack (magenta) moving dorsal to ventral.

Reference brain with Vglut2a expression (green) registered to the ZBB Vglut2a brain (magenta) moving dorsal to ventral.

The average brain across all larvae with glyt2:mCherry expression (green) registered to the ZBB glyt2:GFP brain (magenta) moving dorsal to ventral. Note there are two different transgenic lines used.

The ZBB vglut2a:DsRed stack (magenta) compared with the ZBB inhibitory lines (glyt2:GFP and gad1b:GFP combined, green).

Supplementary information
