# Supplementary material for: Investigation of hindbrain activity during active locomotion reveals inhibitory neurons involved in sensorimotor processing
Source: Sci Rep. 2018 Sep 11;8:13615. doi: 10.1038/s41598-018-31968-4 (PMC6134141; doi:10.1038/s41598-018-31968-4)
Supplement: Supplementary file 10 — Supplementary information [file 41598_2018_31968_MOESM10_ESM.docx]

**Supplementary Information**

**Title of manuscript**

Investigation of hindbrain activity during active locomotion reveals inhibitory neurons involved in sensorimotor processing

**Author list**

Kristen E. Severi^1,2#^, Urs L. Böhm^1,3#^, and Claire Wyart^1*^

**Affiliations**

^1^ Institut du Cerveau et de la Moelle épinière, ICM, Sorbonne Université, Inserm, CNRS, AP-HP, F-75013, Paris, France.

^2^ Present address: Federated Department of Biological Sciences, New Jersey Institute of Technology, University Heights, Newark, NJ 07102, USA.

^3^ Present address: Dept. of Chemistry and Chemical Biology, Harvard University, Cambridge, MA 02138, USA.

^#^ Equal contribution

* Corresponding Author and Lead contact: [claire.wyart@icm-institute.org](mailto:claire.wyart@icm-institute.org)

**Supplementary Figures**


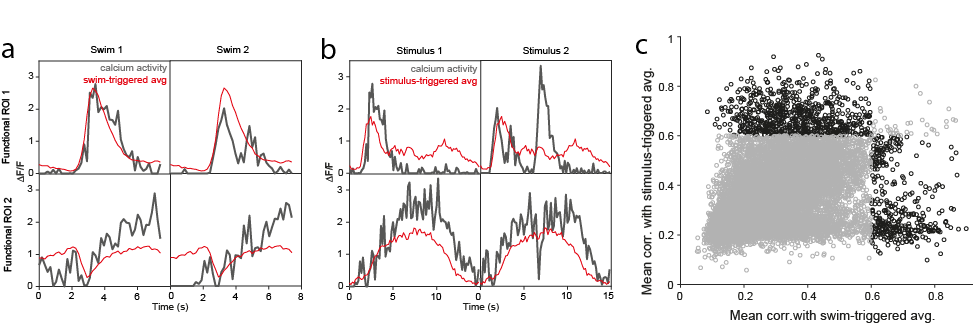


**Supplementary Fig. S1. Related to Figure 2. Selection of ROIs entering the clustering algorithm based on the degree of correlation to visual stimulus or motor output.**

**a,b)** Detail of the two functional ROIs shown in F. ΔF/F calcium trace in shown in grey and swimming- or stimulus-triggered averages are show in red. While individual swim-triggered traces of ROI 1 (**a**) correlate well with its swim-triggered average and not with its stimulus-triggered average, the reverse is true for ROI 2 (**b**).

**c)** Scatter plot of the mean correlation of each ROI with its stimulus-triggered average vs. its swim-triggered average. The grayed-out area corresponds to data excluded from the cluster analysis.


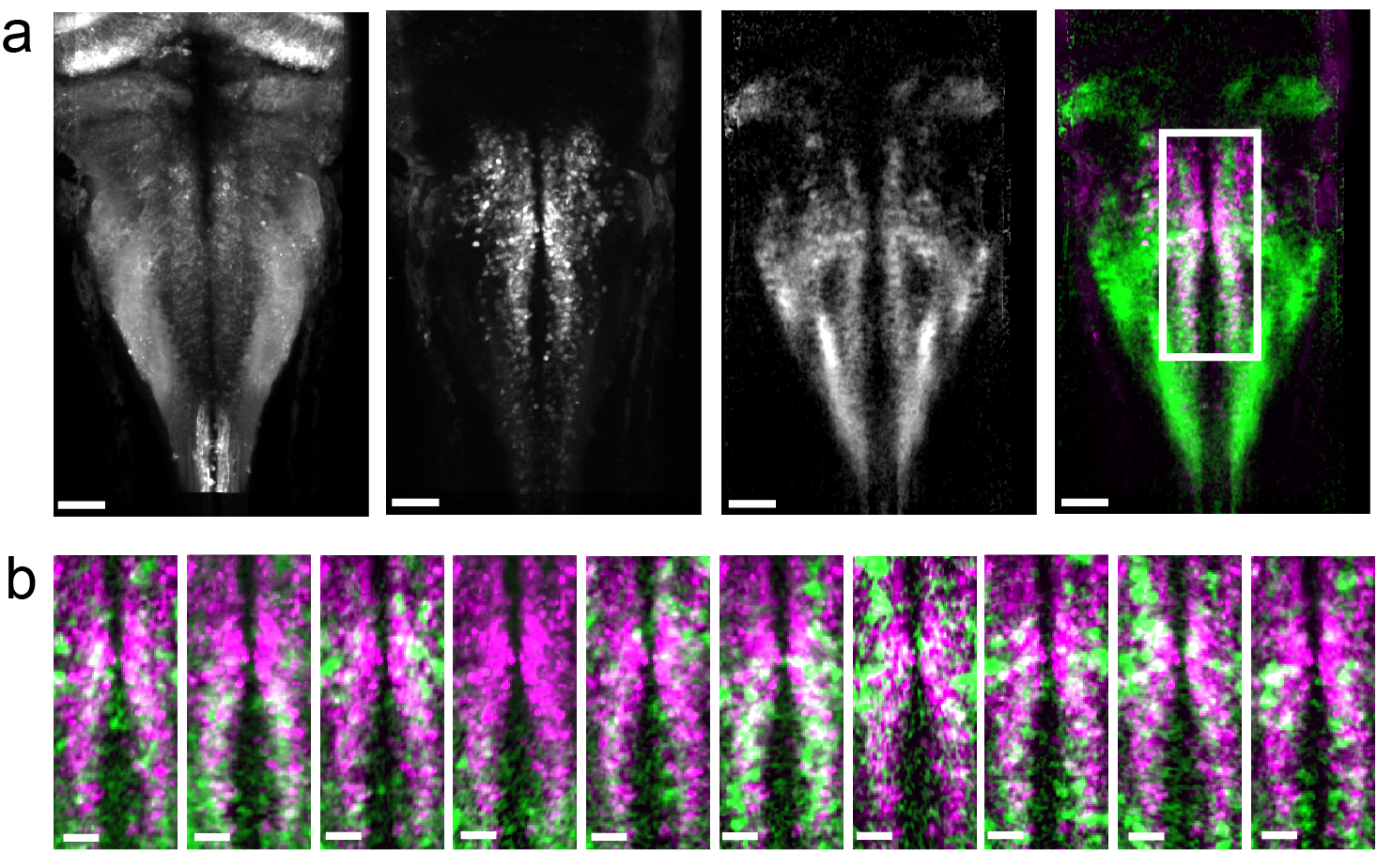


**Supplementary Fig. S2. Related to Figure 3. Overlap between swim cluster pixels and glycinergic neurons in each individual larva.**

**a)** Z-projection of 20 optical sections imaged from the dorsal aspect in a *Tg(HuC:GCaMP5G)* larva showing pan-neuronal localization of GCaMP5G (left); Z-projection of 20 optical sections imaged from the dorsal aspect in a *Tg(glyt2:mCherry)* larva showing localization of mCherry in glycinergic cells (center left); pixels correlated with swim cluster 1 for all 10 larvae in the dataset normalized by the number of larvae contributing to each pixel (center right); overlap between *Tg(glyt2:mCherry)* image (magenta) and swim cluster 1 pixels (green). White box indicates region expanded in (b).

**b)** Same region as (a) and *Tg(glyt2:mCherry)* magenta image same as (a), but swim cluster 1 pixels (green in (a)) displayed for each of the 10 individual larvae which comprise the complete dataset.

Scale bars are 40 µm.


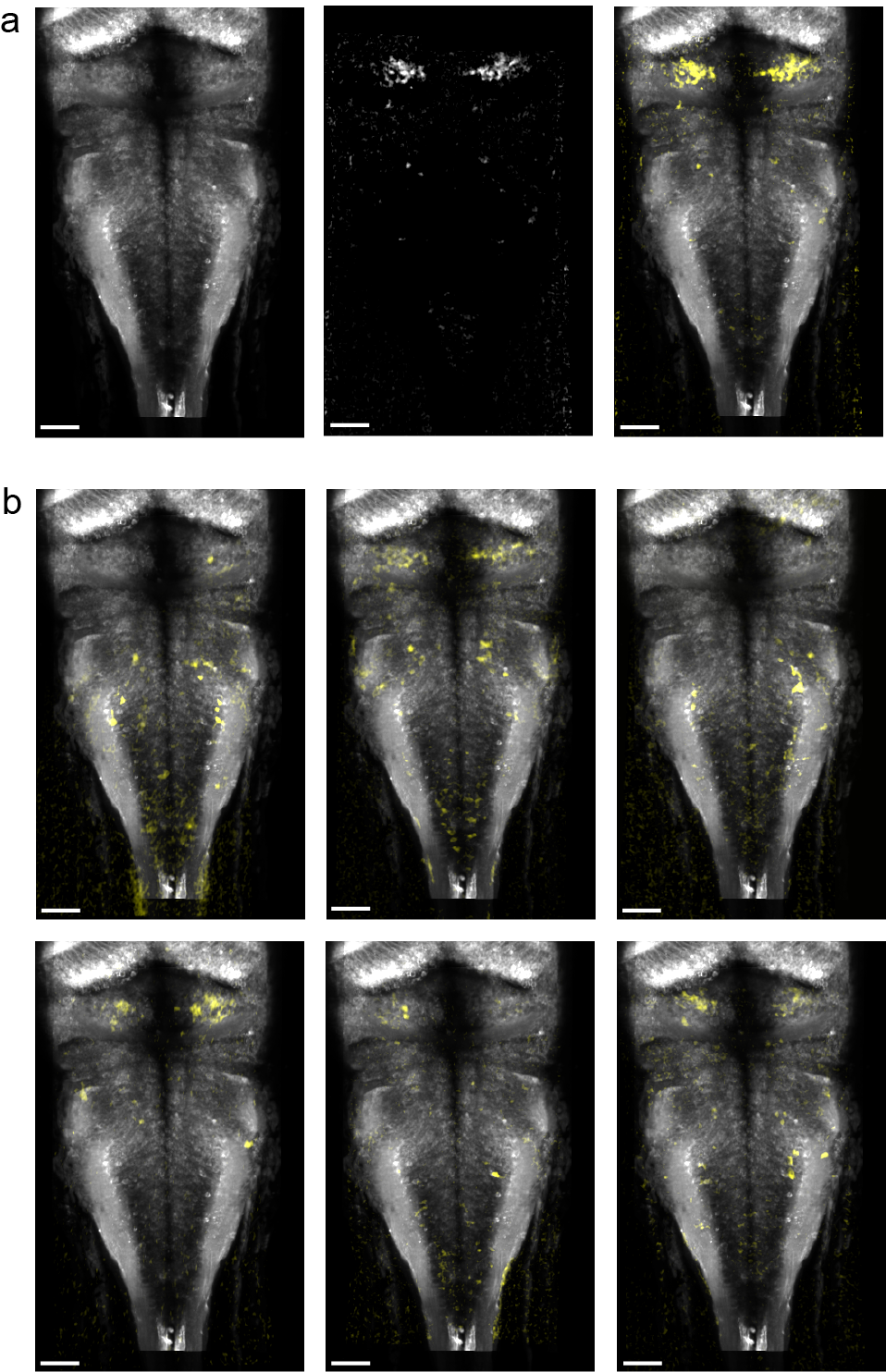


**Supplementary Fig. S3. Related to Figure 4. Visualization of stimulus cluster 3 pixels overlaid with anatomical images of pan-neuronal GCaMP5G expression for all larvae contributing to that cluster.**

**a)** Z-projection stack of 25 optical sections imaged from the dorsal aspect in a *Tg(HuC:GCaMP5G)* larva showing pan-neuronal localization of GCaMP5G (left) or our extracted pixels for stimulus cluster 3 for all 6 larvae where this anatomical region was imaged in the dataset normalized by the number of larvae contributing to each pixel (center). Merge at right with *Tg(HuC:GCaMP5G)* in grey and stimulus cluster 3 pixels in yellow.

**b)** Same region as (**a**) and *Tg(HuC:GCaMP5G)* image (grey) same as (**a**), but stimulus cluster 3 pixels (yellow) separated for the 6 individual larvae which comprise the dataset.

Scale bars are 40 µm.


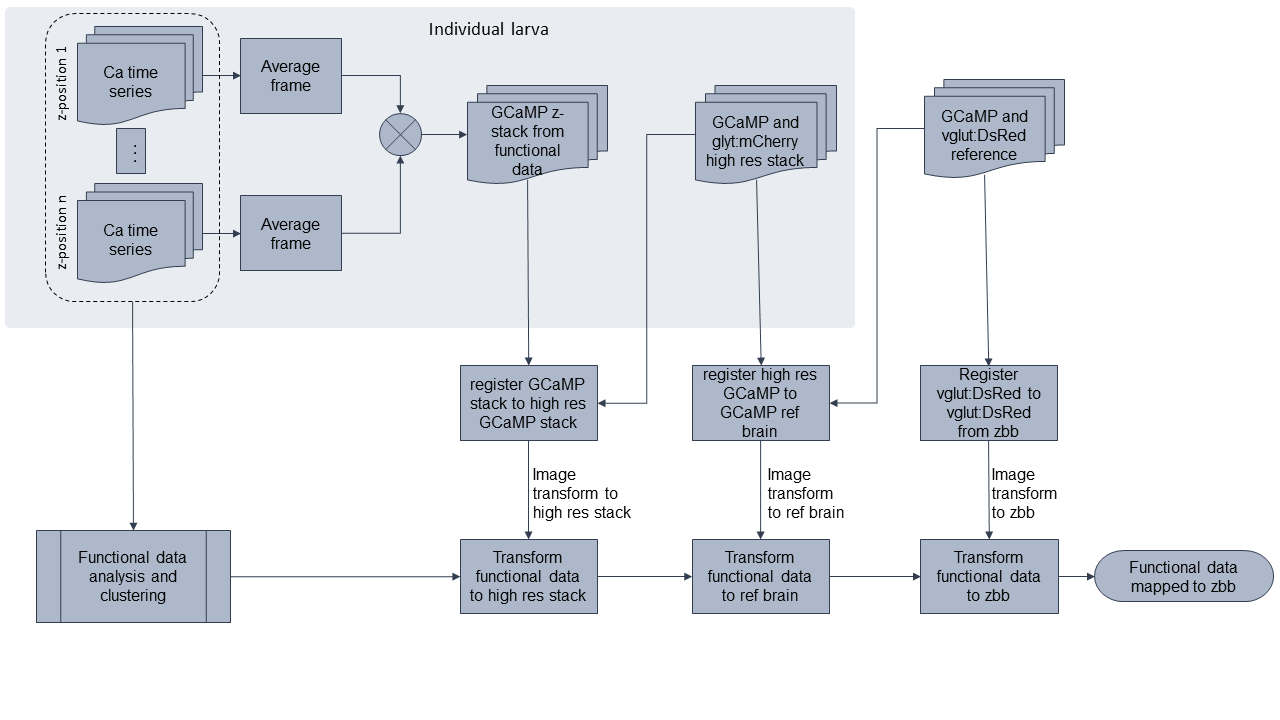


**Supplementary Fig. S4. Related to Methods. Flowchart of data mapping and registration procedure.**

The GCaMP imaging time series from each individual larva are assembled into a z-stack and registered to a high-resolution z-stack of the same larva. The high-resolution GCaMP data of each larva is registered to a high-resolution GCaMP reference brain. The vglut2a:DsRed signal of the reference brain is registered to the vglut2a:DsRed reference brain of the ZBB atlas. The three resulting image transforms are used to map the functional clusters to the ZBB atlas.

**Supplementary Videos**

**Video S1.** Swim cluster 1 pixels (green) and HuC:GCaMP5G (magenta) moving dorsal to ventral.

**Video S2.** Stimulus cluster 1 pixels (green) and HuC:GCaMP5G (magenta) moving dorsal to ventral.

**Video S3.** Stimulus cluster 2 pixels (green) and HuC:GCaMP5G (magenta) moving dorsal to ventral.

**Video S4.** Stimulus cluster 3 pixels (green) and HuC:GCaMP5G (magenta) moving dorsal to ventral.

**Video S5.** Swim cluster 1 pixels (green) and *engrailed1b* (magenta) moving dorsal to ventral.

**Video S6.** HuC:GCaMP5G averaged over all larvae (green) registered to our reference brain HuC:GCaMP5G stack (magenta) moving dorsal to ventral.

**Video S7.** Reference brain with Vglut2a expression (green) registered to the ZBB Vglut2a brain (magenta) moving dorsal to ventral.

**Video S8.** The average brain across all larvae with glyt2:mCherry expression (green) registered to the ZBB glyt2:GFP brain (magenta) moving dorsal to ventral. Note there are two different transgenic lines used.

**Video S9**. The ZBB vglut2a:DsRed stack (magenta) compared with the ZBB inhibitory lines (glyt2:GFP and gad1b:GFP combined, green).
